# The antiplaque/anticariogenic efficacy of *Salvadora persica* (Miswak) mouthrinse in comparison to that of chlorhexidine: a systematic review and meta-analysis

**DOI:** 10.1186/s12903-019-0741-5

**Published:** 2019-04-27

**Authors:** Elaf Jassoma, Lina Baeesa, Heba Sabbagh

**Affiliations:** 1 Faculty of Dentistry, King Abdulaziz University, Jeddah, Saudi Arabia; 2 Department of Orthodontics, Faculty of Dentistry, King Abdulaziz University, P.O. Box 127139, Jeddah, 21352 Saudi Arabia; 3 Pediatric Dentistry Department, Faculty of Dentistry, King Abdulaziz University, P.O. Box 80209, Jeddah, 21589 Saudi Arabia

## Abstract

**Background:**

The plant *Salvadora persica* (miswak) has a long history of use in oral hygiene. Associations between the use of *Salvadora persica* and decreased oral bacteria numbers and plaque scores have been reported. This systematic review and meta-analysis assessed the ability of *Salvadora persica* mouthrinses to reduce plaque/cariogenic bacteria, in comparison to that of chlorhexidine and/or placebo rinses.

**Methods:**

A comprehensive literature search for clinical trials reporting the use of *Salvadora persica* rinses as an antibacterial and/or antiplaque agent in comparison with chlorhexidine and/or placebo rinses was conducted, with no restriction to language. MEDLINE-PubMed, Cochrane Central Register of Controlled Trials, Wiley Online Library, ScienceDirect, and Google Scholar databases were searched to include all articles published up to December 2018. Based on inclusion/exclusion criteria, data were extracted from the identified reports by two independent reviewers. The primary and secondary outcomes measured from the eligible studies were mean plaque scores and mean cariogenic bacterial counts, respectively. Risk of bias of these studies was assessed. A statistical test of homogeneity was used to determine if the results of the separate studies could be combined. Based on the chi-square test, an inconsistency coefficient was computed (I^2^ statistic). Sensitivity analyses using subgroups and homogeneity evaluation were conducted.

**Results:**

A total of 1135 potentially eligible articles were identified, of which 19 were eventually included in the qualitative analysis whereas 18 were included in the quantitative meta-analysis. The meta-analysis showed that *Salvadora persica* rinses exhibited strong antiplaque effects (*P <* 0.00001, MD: 0.46, and 95% CI: 0.29 to 0.63). In addition, it had statistically significant anti-streptococcal (*P <* 0.0001, MD: -1.42, and 95% CI: -2.08 to − 0.76) and anti-lactobacilli effects (*P <* 0.00001, MD: -1.12, and 95% CI: -1.45 to − 0.79) when compared to placebo. However, its effects were inferior compared to those by chlorhexidine rinse (*P* = 0.04, MD: 0.19, and 95% CI: 0.01 to 0.37). Subgroup analyses yielded results similar to those prior to subgrouping.

**Conclusion:**

The use of *Salvadora persica* extract was associated with a significant reduction in the plaque score and cariogenic bacterial count. Although, this reduction was lower than that achieved with the gold standard chlorhexidine mouthwash, *Salvadora persica*-containing rinse could be considered as a suitable oral hygiene alternative for use in individuals of all ages, socioeconomic backgrounds, and health conditions especially as a long-term measure due to its efficacy, safety, availability, cost-effectiveness, and ease of use.

**Electronic supplementary material:**

The online version of this article (10.1186/s12903-019-0741-5) contains supplementary material, which is available to authorized users.

## Background

Dental plaque is considered the major etiological factor for the initiation and progression of the two predominant oral diseases such as dental caries and periodontal disease. These diseases are preventable by regular personal and professional mechanical plaque debridement measures [1].

*Salvadora persica*, also known as miswak or siwak, comes from Arak or the toothbrush tree that grows in west India and Africa [2]. Miswak sticks gained its popularity in Dentistry in 5000 B.C., and were originally used by Babylonians and later used widely throughout other nations [3]. Historically, it has been used in various forms, such as sticks, extract, and toothpaste, for its potent properties. It is still being used globally; in many Muslim countries, it is used in a religious ritual [4], and in developing countries, it is extensively used due to its availability and low cost or as a traditional practice [5]. The effectiveness of this medicinal plant is related to the presence of benzyl isothiocyanate, which is a major component inhibiting acid production and growth of *Streptococcus mutans* [6]. It also has virucidal activity against Herpes simplex virus and is fungistatic against *Candida albicans* [7]. *Salvadora persica* has exhibited the ability to inhibit the adherence of *Streptococcus mutans* to human buccal epithelial cells [8] and has been most sensitive against such bacteria in comparison with other types of bacteria [9]. The inclusion of sulfur and alkaloids (saladorine) has an antibacterial effect and improves gingival health [10]. In addition, tannis forms a protective layer on the enamel against caries, while chloride protects against calculus formation [10]. The plant contains other chemical components, such as tri-methyamin, salvadrin, fluoride, silica, mustard, vitamin C, calcium, and phosphorous [11]. Besides having an antimicrobial effect, *Salvadora persica* has mild laxative, diuretic [10], anti-pyretic, anti-inflammatory, astringent, and analgesic effects [12]**.** The World Health Organization (WHO) has recommended the use of *Salvadora persica* sticks as an effective oral health tool attributed to the mechanical action of the soft wood fibers and the therapeutic action of its chemical constituents [13]. The first reference to mouthrinsing is credited to Chinese medicine, practiced as far back as 2700 B.C. as a treatment for gingival diseases [13]. In more modern times, mouthrinses containing active therapeutic agents have been advocated as vehicles to deliver chemical agents and used as a daily oral health care measure to control dental plaque [14]. Mouthrinses have been shown to be the most feasible, safe, and effective means for achieving an acceptable antimicrobial ecosystem, in conjunction with daily mechanical methods [15].

Chlorhexidine digluconate oral rinse is one of the most common antimicrobials prescribed in the dental field [16]. It has both bacteriostatic and bacteriocidal effects [16] against a variety of micro-organisms including gram-positive and gram-negative aerobic and anaerobic bacteria [17] as well as fungi including yeasts [18]. Chlorhexidine is regarded as the gold standard due to its substantivity effects and high antimicrobial activity. Substantivity refers to its ability to be continually released into the oral cavity and adhere persistently to oral tissues [19].

It has been demonstrated that *Salvadora persica*-containing rinses inhibited the growth of cariogenic bacteria [9, 20, 21]. In this regard, Al-Dabbagh et al. (2016) compared the antimicrobial effects of miswak mouthwash, miswak toothpaste, and ordinary toothpaste, and found that the miswak products, especially mouthwashes, were more effective in reducing the growth of cariogenic bacteria than that by ordinary toothpaste [22]. Numerous studies have established that *Salvadora persica* extracts are effective in reducing plaque accumulation. However, in terms of which antimicrobial agent being regarded as more effective, some studies indicated that *Salvadora persica* and chlorhexidine rinses showed similar antiplaque effects [23–27], while others favored the use of chlorhexidine [28–34]. On the other hand, interestingly, two studies had reported that the mean plaque index of *Salvadora* persica was significantly lower than that observed with chlorhexidine [35, 36].

Previous studies showed inconsistent and dissimilar findings as to which antimicrobial mouthwash is more effective in reducing plaque scores. Further, no meta-analysis has been previously performed on the effects of *Salvadora persica* versus chlorhexidine. Gaining more insight into the properties of *Salvadora persica* may pave the way for its use as an alternative for plaque control and caries prevention, particularly in communities with low socioeconomic status, where it is more conveniently and easily obtained. Therefore, the aim of this systematic review and meta-analysis was to assess the use of *Salvadora persica*-containing mouthrinses among healthy individuals in comparison to the gold standard (chlorhexidine gluconate mouthrinse) and/or placebo rinses in terms of decreasing both plaque and cariogenic microbial load.

## Method

### Registration

This systematic review has been registered on PROSPERO (https://www.crd.york.ac.uk/prospero/); register #CRD42018094678.

### Search strategy

This comprehensive systematic review included in-vivo studies reporting the use of *Salvadora persica* mouthrinse in comparison to chlorhexidine and/or placebo, in terms of its anticariogenic and/or antiplaque effects. The search strategy comprised keywords that were listed either separately or in combination with other words: ((Miswak) OR (herbal) OR (Siwak) OR (Arak) OR (peelu) OR (Salvadora persica)) AND ((chlorhexidine)) AND ((mouthwash) OR (mouthrinse)) AND ((antimicrobial) OR (antibacterial) OR (antiplaque)). All publications in MEDLINE-PubMed, Cochrane Central Register of Controlled Trials, Wiley Online Library, ScienceDirect, and Google Scholar were reviewed by two assessors (EJ and LB) independently. The search was done in December 2018 and sought to identify all articles related to the topic that had been published up to that date, with no constraints in terms of language. Moreover, the references of the identified studies were reviewed manually. Authors of four studies were contacted via email to acquire additional data that were not available in the published articles.

### Screening process

Two authors (EJ and LB) reviewed the titles and abstracts of all articles independently. Any disagreement among authors were settled in consensus meetings and in discussion with a third author (HS). After consensus, the three reviewers (EJ, LB, and HS) retrieved the full-text article for screening and data extraction. The references of the remaining articles were reviewed manually.

### Eligibility criteria

Papers were screened according to the following *inclusion criteria*:

- Population (P): clinical trials and in-vivo studies conducted on medically healthy individuals;
- Intervention (I): the use of *Salvadora persica* mouthwash;
- Comparison (C): the use of chlorhexidine mouthwash and/or placebo; and
- Outcome (O): a decrease in the mean plaque score and cariogenic bacterial counts.

The *exclusion criteria* were as follows:

- Reviews of the literature, case–control, cross-sectional, and in-vitro studies;
- Use of *Salvadora persica* sticks or toothpaste;
- Use of mechanical methods for plaque removal;
- Patients with periodontal breakdown and active caries;
- Patients with orthodontic wires/brackets; and
- Studies of the effects of *Salvadora persica* on other types of bacteria.

The following data were extracted by two of the authors (EJ and LB):

- The study design and setting;
- Total sample size, samples allocated per group, and sample description;
- Eligibility criteria for including participants;
- Baseline and post-treatment measurements of the intended outcomes in *Salvadora persica* mouthwash users versus other interventions;
- The concentration of *Salvadora persica* used;
- The frequency and duration of the interventions; and
- The plaque index system used.

### Risk of bias assessment

The quality of the studies that fulfilled the inclusion criteria were assessed according to the CONSORT 2010 checklist [37] used for randomized trials. The scale measured two items: methods and results, which included the trial design, participants, participant flow, interventions, outcomes, sample size, randomization, blinding, recruitment, baseline data, numbers analyzed, ancillary analyses, and harms, with a minimum score of 0 and a maximum score of 27. Studies with scores of 9 or less were regarded as being of low quality; 10–18 were considered to be of moderate quality; and studies with a score of 19 or more were considered as being of high quality. The details of this assessment is listed in Additional file 1: Table S1. As for the risk of bias, the included studies were assessed against the five main domains: selection bias, performance bias, attrition bias, reporting bias, and other sources of bias, in accordance with methods recommended by the Cochrane Handbook for Systematic Reviews of Interventions [38]. The following judgments were used: low risk, high risk, or unclear (either for the lack of information or uncertainty over the potential for bias). The details of this evaluation along with explanation and comments are listed in Additional file 2: Table S2. The quality assessment, as well as the risk of bias assessment, were evaluated by two review authors (EJ and LB) independently. Authors resolved any disagreement by consensus, and the third author (HS) was consulted to resolve any conflict if necessary.

Moreover, the strength of evidence and recommendation was evaluated using Shekelle and colleagues system [39].

### Statistical analysis

The meta-analysis was carried out using the free Review Manager software (Cochrane Collaboration). A statistical test of homogeneity was implemented in order to determine whether the results of the separate studies could be combined. An inconsistency coefficient was calculated (I^2^ statistic), which was based on the chi-square test. If the value was 50% or more, it signified that the results of such studies were of moderate heterogeneity, whereas a value of 75% or more signified the presence of high heterogeneity between the studies [38]. Odd ratios with a fixed effect model was used for homogenous studies, whilst a random effect model was used for the heterogeneous studies. Forest plots were used to display mean differences (MD) and their 95% confidence interval (CI) of individual studies and a summary estimate of effect.

### Sensitivity analysis

Subgroup analyses were conducted to assess the stability of the studies. Papers that assessed the antiplaque effects of the mouthrinses were sub-grouped according to the concentration of chlorhexidine used, duration of mouthwash use, refraining of mechanical plaque measures, and strength of the included studies. The duration of mouthwash used was sub-divided into 3-week intervals. If the results of these analyses were similar to those performed prior to subgrouping, then it is unlikely to be the source of heterogeneity. Funnel plots were used to assess precision and heterogeneity as a consideration of possible small study effects.

## Results

### Systematic review results

This revised search strategy yielded a total of 1135 potentially eligible articles that were published between January 1970 and December 2018. The results generated across the five databases were as following: MEDLINE-PubMed (77), Cochrane Central Register of Controlled Trials (28), Wiley Online Library (193), ScienceDirect (164), and Google Scholar (674). From these, 123 duplicates were excluded. The titles of the remaining 1012 articles were evaluated. Subsequently, 935 articles were eliminated after the titles and abstracts had been assessed. The full texts of the remaining 77 papers were reviewed and compared to the inclusion criteria chosen.

Fifty-nine articles were excluded for the following reasons: because they were not in-vivo studies (four articles), did not involve periodontally healthy participants (nine articles), did not use *Salvadora persica* products/rinses (38 articles), or did not include an applicable comparison group (eight articles). However, there were no studies that compared *Salvadora persica* with chlorhexidine as an antibacterial agent.

**Fig. 1:**
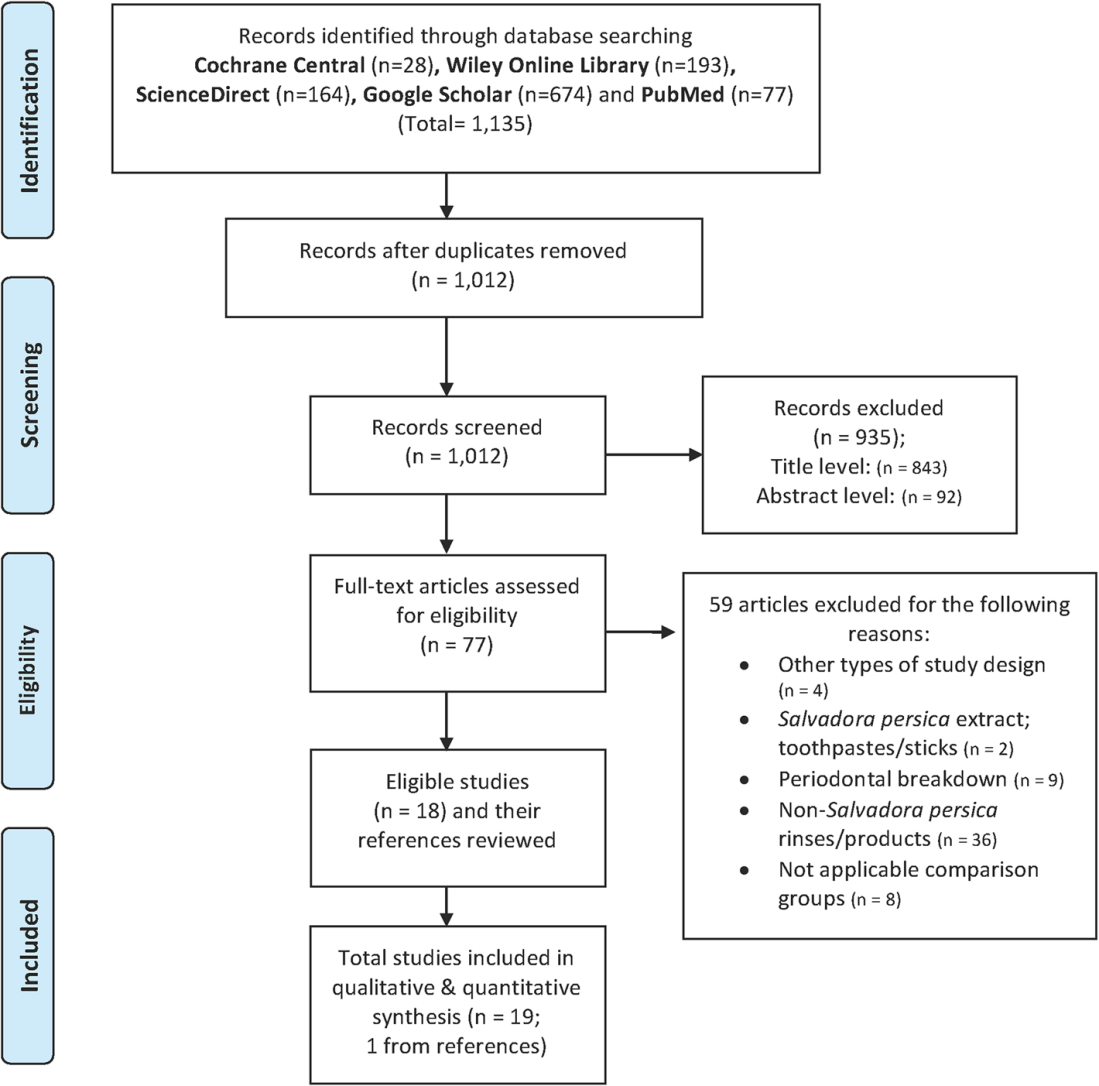
Flow chart of study selection process

The search strategy was conducted according to the PRISMA 2009 guidelines [40], and yielded 18 eligible articles (Fig. 1). One other article was added from the reference lists of eligible articles. Thus, 19 articles were included in the qualitative analysis, all of which were on clinical trials discussing the antiplaque (15 articles), antibacterial (three articles), or both (one article) effects of *Salvadora persica* mouthrinses in comparison to either chlorhexidine or placebo. Non-randomized studies (one article) were excluded from the quantitative analysis to ensure the validity of the results [28]. Hence, 18 articles were included in the quantitative synthesis. Kappa score showed 100% perfect agreement between authors in the search strategy and which article to include or exclude from the analysis. We were able to obtain the mean and standard deviation (SD) values that were not available in one published study directly from the corresponding author [32].

Additional file 3: Table S3 summarizes the characteristics of the articles that were included in the meta-analysis. The most common preparation used was a solution containing 7% *Salvadora persica* extract [23, 25, 26, 29, 30, 32–34, 36, 41]. The studies were mostly performed in adult patients, up to 60 years old, whereas five studies were performed in children between the ages of 6 and 18 years [20–22, 27, 29, 36]. Some studies were of cross-over nature, with a washout period ranging from 2 to 10 days [28, 31, 35] and 2 to 8 weeks [9, 23, 29]. The inclusion and exclusion criteria of the eligible studies are listed in Additional file 4: Table S4.

### Meta-analysis results

**Fig. 2:**
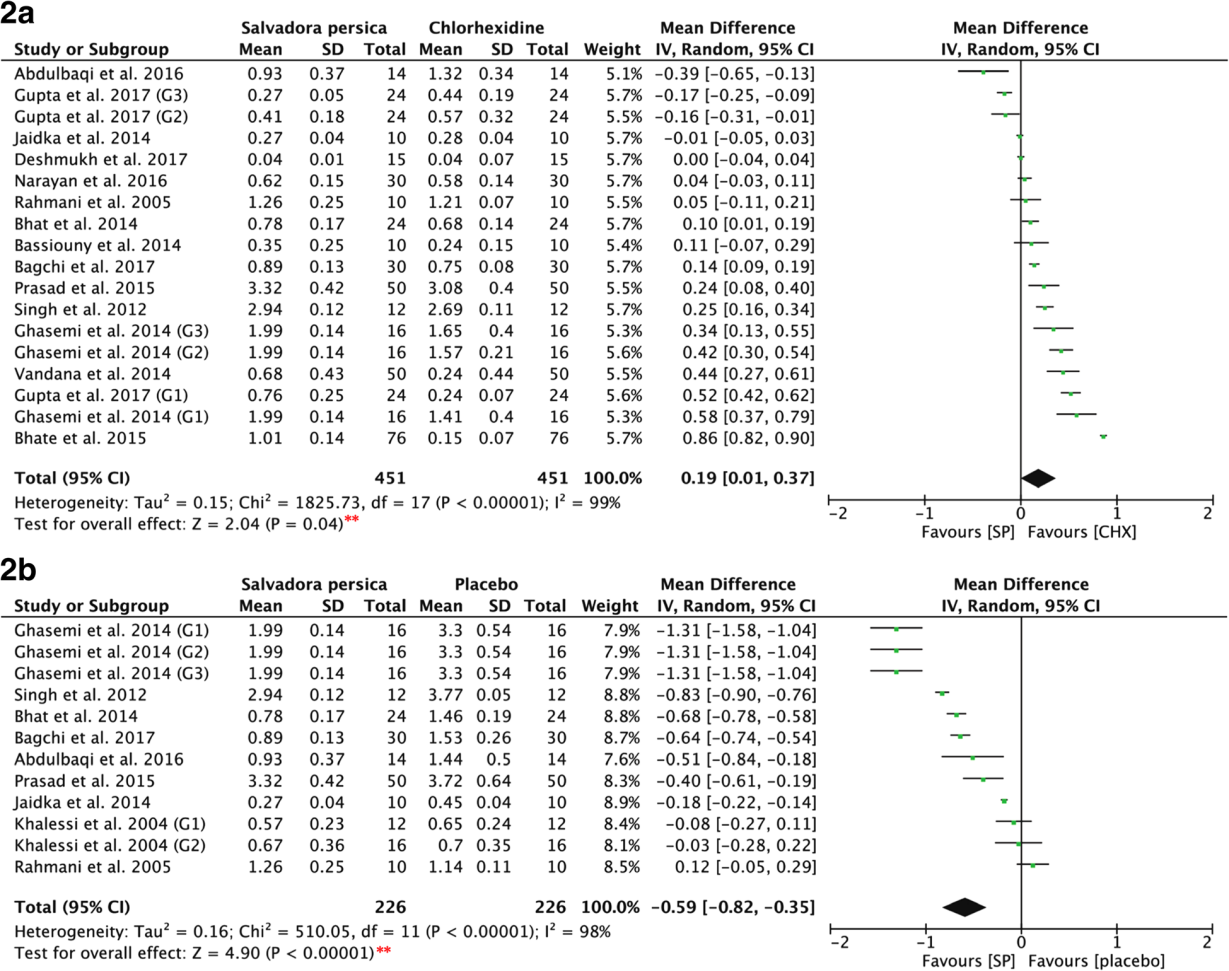
**2a**. Forest plot for meta-analysis of the post-intervention mean plaque scores of *Salvadora persica* in comparison to both concentrations (0.12% & 0.2%) of chlorhexidine. **2b**. Forest plot for meta-analysis of the post-intervention mean plaque scores of *Salvadora persica* in comparison to placebo. [*SP* Salvadora persica, *CHX* chlorhexidine, **: significant difference at *p* = 0.05]

Studies measuring the ability of *Salvadora persica* to reduce the mean plaque scores were assessed. Fig. 2a shows that there was a significant difference in the mean plaque scores of *Salvadora persica* as compared to chlorhexidine (*P* = 0.04, MD: 0.19, and 95% CI: 0.01 to 0.37). Furthermore, the post-intervention mean plaque scores of *Salvadora persica* (Fig. 2b) showed statistically significant differences as compared to placebo (*P* < 0.00001, MD: -0.59, and 95% CI: -0.82 to − 0.35).

Figure 32c shows the pre- and post-intervention values of all three mouthrinses: *Salvadora persica*, chlorhexidine, and placebo rinses demonstrated significant differences (*P* = 0.00001). *Salvadora persica* showed significant antiplaque effects as compared to the pre-intervention scores (*P* < 0.00001, MD: 0.46, and 95% CI: 0.29 to 0.63). Further, both 0.12 and 0.2% chlorhexidine significantly reduced plaque accumulation post-rinsing (*P* < 0.00001, MD: 1.79, and 95% CI: 1.39 to 2.19; and *P* < 0.0006, MD: 0.45, and 95% CI: 0.19 to 0.70, respectively). Placebo rinses showed nonsignificant differences between pre- and post-intervention (*P* = 0.07, MD: -0.19, and 95% CI: -0.39 to 0.02).

**Fig. 3:**
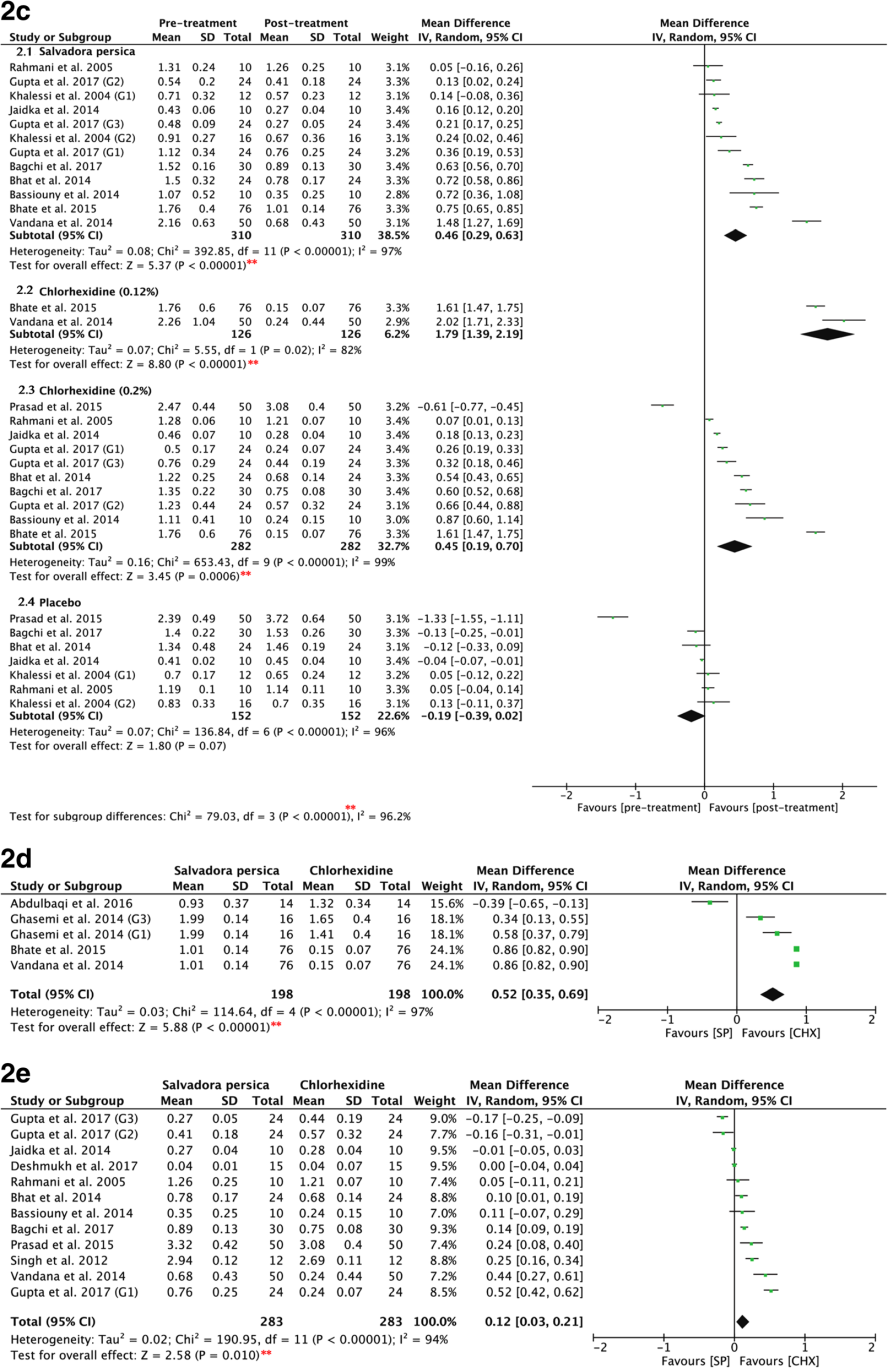
**2c**: Forest plot for meta-analysis of the pre- and post-treatment mean plaque scores of: (2.1) *Salvadora persica*, (2.2) 0.12% chlorhexidine, (2.3) 0.2% chlorhexidine, and (2.4) placebo rinse. **2d**: Forest plot for meta-analysis of the post-intervention mean plaque scores of *Salvadora persica* in comparison to 0.12% of chlorhexidine (sub-grouped according to concentration of chlorhexidine). **2e**: Forest plot for meta-analysis of the post-intervention mean plaque scores of *Salvadora persica *in comparison to 0.2% of chlorhexidine (sub-grouped according to concentration of chlorhexidine). [*SP* Salvadora persica, *CHX* chlorhexidine, **: significant difference at *p* = 0.05]

Studies measuring the ability of *Salvadora persica* to reduce cariogenic bacterial loads were also assessed. The use of *Salvadora persica*-containing rinses resulted in significant decreases in both *Streptococcus mutans* (Fig. 43a) and *Lactobacillus* counts (Fig. 43d) as compared to the use of placebo rinses (*P* < 0.0001, MD: -1.42, and 95% CI: -2.08 to − 0.76; and *P* < 0.00001, MD: -1.12, and 95% CI: -1.45 to − 0.79, respectively).

**Fig. 4:**
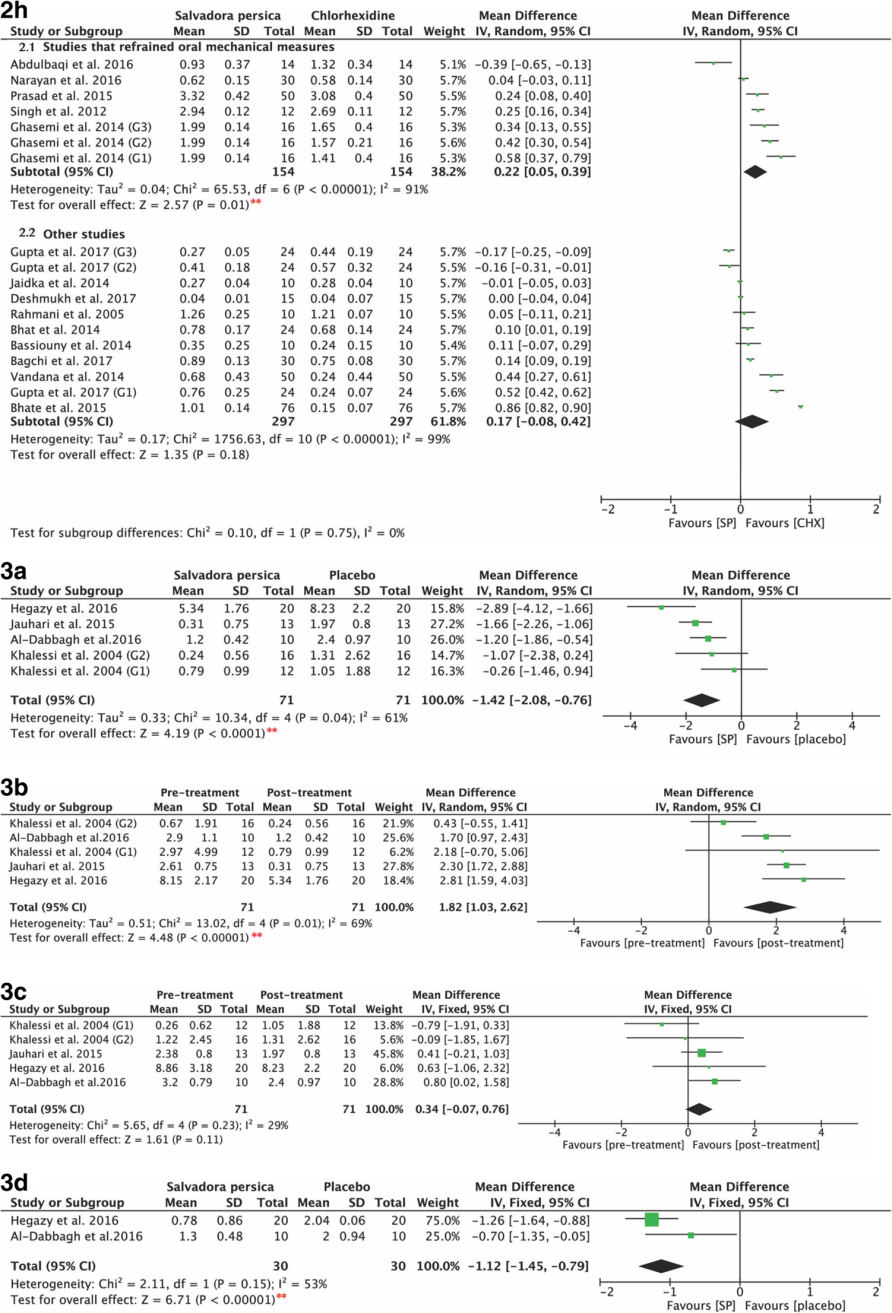
**2h** Forest plot for meta-analysis of the post-intervention mean plaque scores of *Salvadora persica* compared to chlorhexidine (sub-grouped according to refrain of oral mechanical measures); (2.1) studies that refrained mechanical measures and (2.2) studies that did not refrain such measures. **3a** Forest plot for meta-analysis of post- treatment *Streptococcus mutans* counts of *Salvadora persica* in comparison to placebo. **3b** Forest plot for meta-analysis of the pre- and post-treatment *Streptococcus mutans* counts of *Salvadora persica*. **3c** Forest plot for meta-analysis of the pre- and post-treatment *Streptococcus mutans* counts of placebo rinses. **3d** Forest plot for meta-analysis of post-treatment *Lactobacilli* counts of *Salvadora persica* in comparison to placebo. [*SP* Salvadora persica, *CHX* chlorhexidine, **: significant difference at *p* = 0.05]

Figure 43b demonstrates the post-treatment values for the anti-streptococcal effects of *Salvadora persica* and placebo. The use of *Salvadora persica*-containing rinses resulted in statistically significant differences in post-treatment scores as opposed to the pre-treatment values (*P <* 0.00001, MD: 1.82, and 95% CI: 1.03 to 2.62). It had similar effects in reducing lactobacillus counts (*P <* 0.00001, MD: 1.54, and 95% CI: 0.91 to 2.17), as shown in Fig. 53e.

**Fig. 5:**
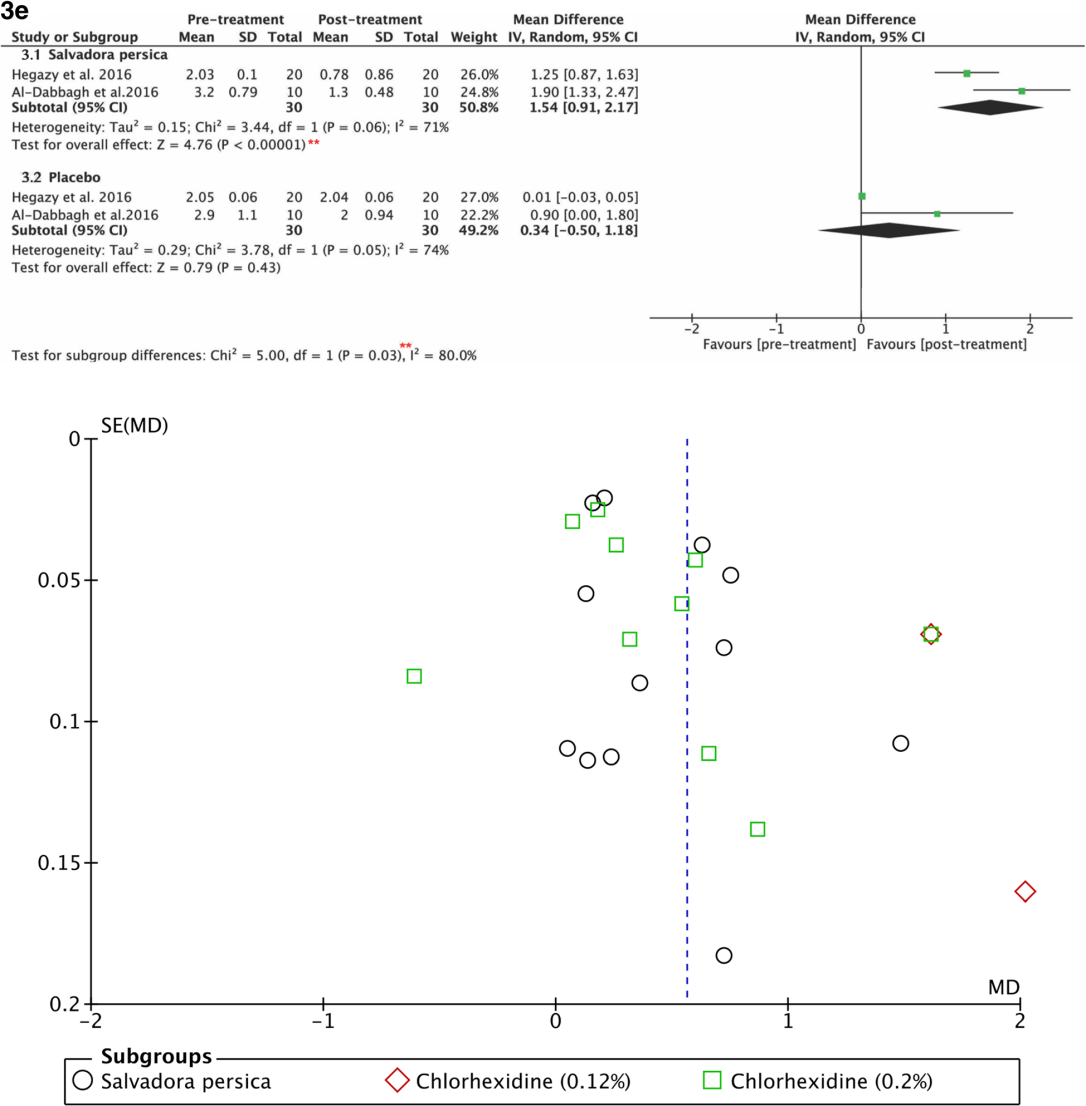
**3e** Forest plot for meta-analysis of the pre- and post-treatment *Lactobacilli* counts of: (3.1) *Salvadora persica* and (3.2) placebo. **: significant difference at *p*=0.05. Funnel plot for meta-analysis of the pre- and post-treatment mean plaque scores of: *Salvadora persica*, 0.12 % chlorhexidine and 0.2% chlorhexidine

### Assessing risk of bias

**Fig. 6:**
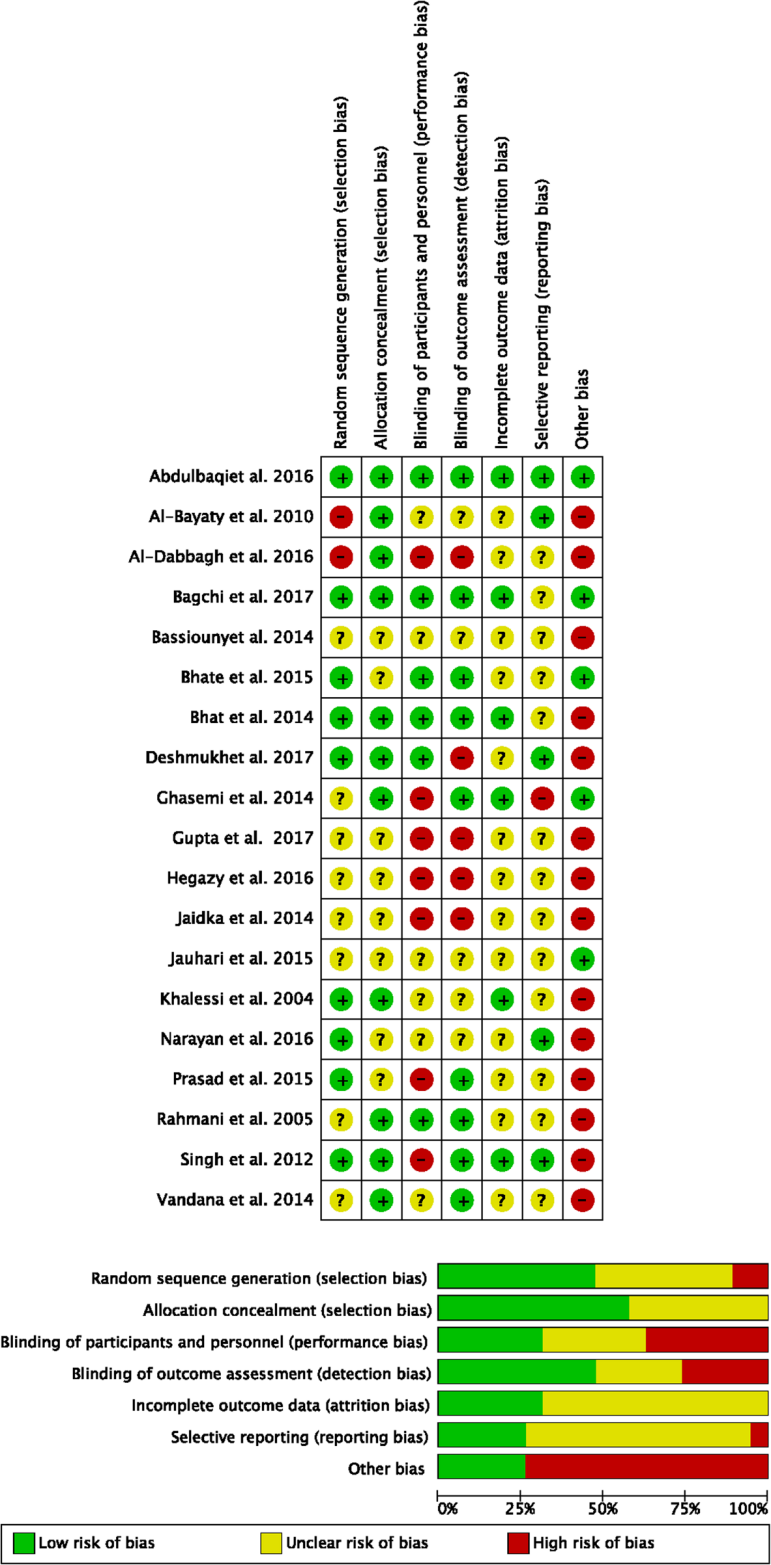
Risk of bias: review authors' judgements about each risk of bias item presented as percentages and as a summary

Although the quality of studies varied due to the use of different criteria, none were excluded based on quality level. Seven papers were regarded as high quality [25, 31, 32, 34, 35, 41], while the remaining papers were of moderate quality [9, 20–24, 26–30, 36]*.* As for the risk of bias,

Figure 6 shows the author’s judgments about each risk of bias item for each included study presented as percentages and as a summary. Additional file 2: Table S2 presents a more detailed appraisal of the risk of bias that includes the authors’ assessment and comments.

Since a non-randomized study was excluded from the quantitative analysis [28], the grade of evidence was Ia with a strength of recommendation being class A for both the antiplaque and antibacterial effects, according to the system of Shekelle and colleagues.

### Sensitivity analysis

There was statistical heterogeneity in the pooled effect estimates. The subgrouping results according to the concentration of chlorhexidine used were similar to those prior to subgrouping, indicating that the result of the meta-analysis showed stability and reliability (Fig. 32d, 32e). The results showed significant difference favoring the use of both 0.12 and 0.2% of chlorhexidine over *Salvadora persica* (*P <* 0.00001, MD: 0.52, and 95% CI: 0.35 to 0.69; and *P* = 0.010, MD 0.12, and 95% CI: 0.03 to 0.21, respectively). A similar conclusion is drawn when subgrouped according to the quality of the study (Fig. 72f) in which high-quality studies favored the use of chlorhexidine (*P =* 0.003, MD: 0.19, and 95% CI: 0.06 to 0.31). When the data were subgrouped according to the duration of mouthwash use (Fig. 72g), chlorhexidine exhibited superior effects when used for less than 3 weeks (*P =* 0.03, MD: 0.15, and 95% CI: 0.01 to 0.29), whereas its long-term use showed similar efficacy in relation to *Salvadora persica* in preventing plaque formation (*P* = 0.18, MD: 0.24, and 95% CI: -0.11 to 0.60). In Fig. 42h, data are subgrouped according to whether the participants were asked to refrain from their daily oral mechanical measures of plaque control. Chlorhexidine demonstrated greater antiplaque effects in the absence of other plaque control measures (*P =* 0.01, MD: 0.22, and 95% CI: 0.05 to 0.39).

**Fig. 7:**
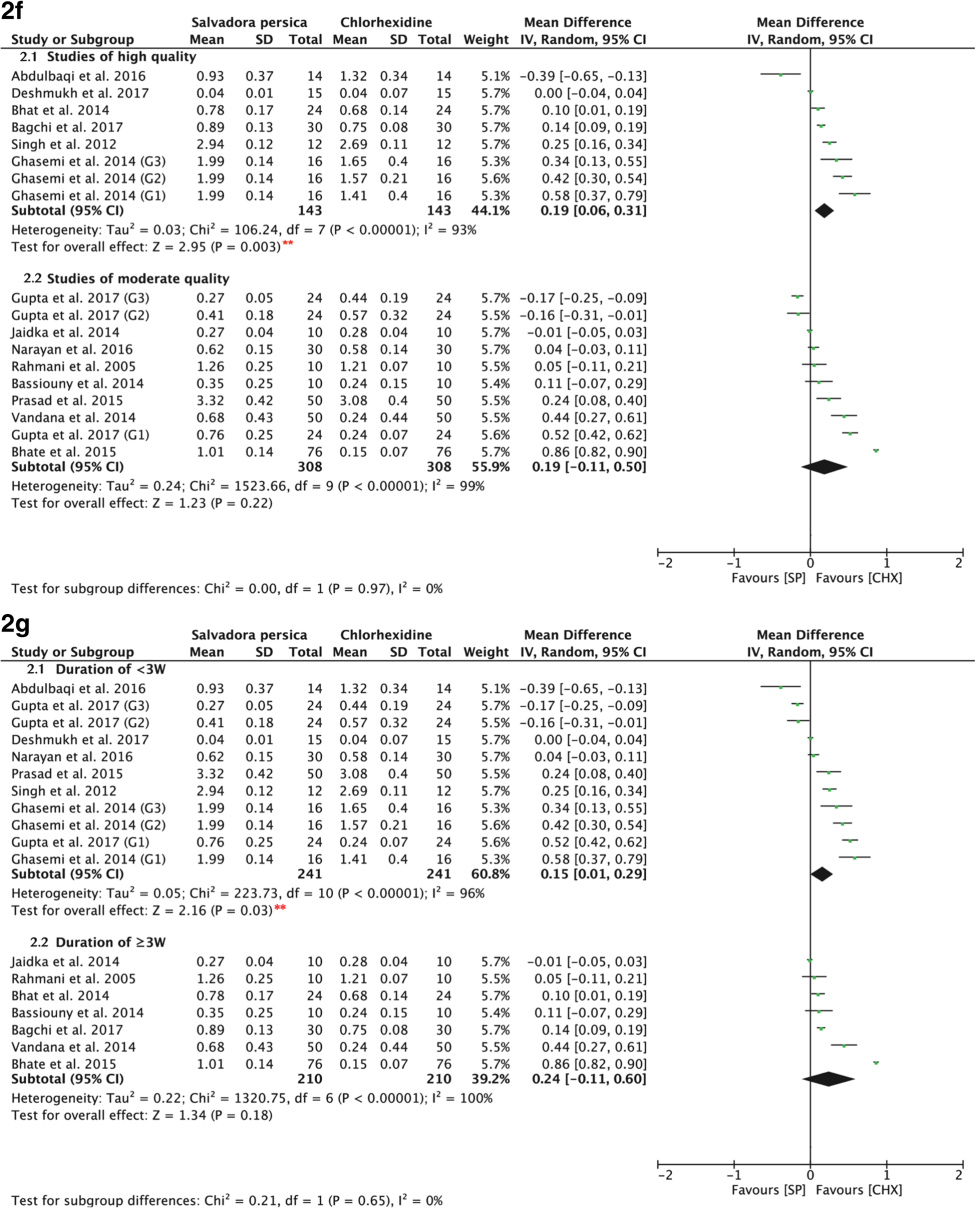
**2f** Forest plot for meta-analysis of the post-intervention mean plaque scores of *Salvadora persica* compared to chlorhexidine (sub-grouped according to risk of bias assessment); (2.1) high quality studies (2.2) moderate quality studies. **2g** Forest plot for meta-analysis of the post-intervention mean plaque scores of *Salvadora persica* compared to chlorhexidine (subgrouped according to duration); (2.1) duration of < 3 weeks and (2.2) duration of ≥3 weeks. [*SP* Salvadora persica, *CHX* chlorhexidine, **: significant difference at *p* = 0.05]

However, subgrouping according to other variables and subgrouping studies assessing anticariogenic bacterial effects were not possible because of the limited number of studies. The funnel plot of the studies at pre- and post-intervention plaque scores of the three mouthrinses exhibited scattering indicating heterogenity (Fig. 5).

## Discussion

With the advancement in the field of dentistry, the focus is leaning more towards preventive measures that target the causative factors of oral diseases. The control of plaque accumulation is, therefore, of primary importance specifically using natural antimicrobial mouthrinses to complement and synergistically aid mechanical plaque removal. Since the most commonly used herbal plant is *Salvadora persica*, the emphasis of the current study was to investigate its efficacy in comparison to chlorhexidine.

The present meta-analysis results indicate that extracts of *Salvadora persica* significantly inhibited plaque formation and yielded mean plaque index scores lower than that of placebo. However, its effectiveness is inferior compared to either 0.12% or 0.2% of chlorhexidine. Such findings are in accordance with several other studies [28–30]. There is no evidence that one concentration of chlorhexidine is more effective than the other in reducing gingivitis and plaque [42]. This could explain why *Salvadora persica* rinses are less effective than either concentration of chlorhexidine. In addition, different concentrations of chlorhexidine can achieve similar effects when the correct volume is used. Larger volumes of a low concentration (0.12%) can be just as effective as smaller volumes of a high concentration (0.2%) of chlorhexidine [43]. The antiplaque effects of chlorhexidine and *Salvadora persica* were not statistically significant when mechanical plaque measures were used. Therefore, in order to gain maximum benefits of using *Salvadora persica* mouthrinses, it should be supplemented with regular mechanical plaque control such as brushing.

Studies that used a cross-over design were included in the meta-analysis, in which each of its trial periods was added to the quantitative analysis as an individual study. The established substantivity effect of chlorhexidine has been reported to be 12–14 h [19] whereas, *Salvadora persica* extracts had a residual effect lasting to 3 h post-rinsing [31]. Since the wash-out periods of the studies were no less than 2 days, the wash-out phase between the trial periods was considered sufficient to rule out a carry-over effect of the assigned mouthwashes.

Once long-term use of mouthrinse is needed, chlorhexidine substitutes should be considered due to the adverse effects that have been associated with its long-term use such as discoloration of teeth and some restorations, alteration in taste sensation [38], stomatitis, ulceration/erosions [39], sloughing off of the oral mucosa, supragingival calculus formation [40], and sensation of tongue burning [41], amongst others. For this reason, not only did the Food and Drug Administration of the USA recommend limiting the use of chlorhexidine rinses to 6 months (https://www.drugs.com/pro/chlorhexidine.html) but also the World Health Organization (WHO) advised to investigate the possible use of natural plants and herb extracts [13, 28]. Since the result of subgrouping according to duration indicate similar antiplaque properties of *Salvadora persica*-containing rinses and chlorhexidine when used for a period exceeding 3 weeks, *Salvadora persica* rinses can be considered as a viable alternative in refractory cases and as a daily rinse when desired. Additionally, *Salvadora persica* mouthwashes are suitable for children and individuals with special needs who run the risk of accidentally ingesting chemically formulated mouthrinse solution or fluoridated toothpaste. It is also an affordable choice for patients of low socio-economic status. Collectively, *Salvadora persica* rinses is an option that can improve the oral health-related quality of life of individuals of different ages, socioeconomic background, and medical conditions.

## Limitations

The heterogeneity observed between studies might have resulted from different methodologies followed, study designs, and small sample sizes in the individual studies. Heterogeneity was overcome by the use of random effects instead of fixed effects analysis. In addition, whenever possible, subgroup analysis was performed for factors considered to be sources of heterogeneity.

## Conclusion and recommendations

This meta-analysis and systematic review indicated clear antiplaque and anticariogenic effects of *Salvadora persica* extracts with a recommendation evidence of Ia and class A strength [39]. Although its effects are considered inferior to the gold standard chlorhexidine, it is still an effective antimicrobial that can be used as an alternative, especially for long-term use. Future high-quality randomized controlled clinical trials with larger sample sizes and longer follow-up periods are recommended, particularly in communities with low-income patients and medically compromised children. Additionally, different concentrations of *Salvadora persica* should be studied in-vivo in order to reach consensus about the most effective concentration of *Salvadora persica* to use for reducing different oral microbes.

## Additional files

Additional file 1: **Table S1.** Quality assessment according to CONSORT (2010) checklist. The scale measured two items: methods and results, which included the trial design, participants, participant flow, interventions, outcomes, sample size, randomization, blinding, recruitment, baseline data, numbers analyzed, ancillary analyses, and harms. (XLSX 32 kb)

Additional file 2: **Table S2.** Risk of bias assessment according to the modified Cochrane Collaboration tool for randomized controlled trials. The details of the risk of bias assessment along with explanation and comments. Assessments were done according to five main domains: selection bias, performance bias, attrition bias, reporting bias, and other sources of bias. (XLSX 20 kb)

Additional file 3: **Table S3.** Characteristic of the included study; type of study, date, location, age group, sample size, frequency of intervention, duration, plaque index system used, means and standard deviations, amount and percentage of intervention, and *P* value. (XLSX 48 kb)

Additional file 4: **Table S4.** The inclusion and exclusion criteria of each study. (XLSX 36 kb)

## Abbreviations

CI: Confidence interval
MD: Mean difference

## References

[1] Technical WHO, Note I, Facts KEY (1944). Sugars and dental caries. J Public Health Dent.

[2] Ezoddini-Ardakani F (2010). Efficacy of Miswak (salvadora persica) in preventing dental caries. Sci Res.

[3] Almas AK, Almas K (2013). Miswak (salvadora persica chewing stick) and its role in oral health; an update. JPDA..

[4] Haque MM, Alsareii SA (2015). A review of the therapeutic effects of using miswak (Salvadora Persica) on oral health. Saudi Med J..

[5] Wu CD, Darout IA, Skaug N (2001). Chewing sticks: timeless natural toothbrushes for oral cleansing. J Periodontal Res.

[6] Lucchini JJ, Corre J, Cremieux A (1990). Antibacterial activity of phenolic compounds and aromatic alcohols. Res Microbiol.

[7] Sofrata A, Brito F, Al-Otaibi M, Gustafsson A (2011). Short term clinical effect of active and inactive Salvadora persica miswak on dental plaque and gingivitis. J Ethnopharmacol.

[8] Hammad M, Sallal A-K (2005). Inhibition of Streptococcus mutans. Adhesion to buccal epithelial cells by an aqueous twigs extract of Salvadora persica. Pharm Biol.

[9] Khalessi AM, Pack AR, Thomson WM, Tompkins GR (2004). An in vivo study of the plaque control efficacy of Persica: a commercially available herbal mouthwash containing extracts of Salvadora persica. Int Dent J.

[10] Bismelah NA b, Kassim ZHM, Ahmad R, Ismail NH (2016). Herbs in dentistry.

[11] Moher D, Hopewell S, Schulz KF, Montori V, Gøtzsche PC, Devereaux PJ (2010). Reporting CONSORT 2010 explanation and elaboration: updated guidelines for reporting parallel group randomized trials. BMJ..

[12] Ahmad H, Rajagopal K (2013). Biological activities of *Salvadora persica L*. (Meswak). Med Aromat Plants.

[13] Mandel ID (1988). Chemotherapeutic agents for controlling plaque and gingivitis. J Clin Periodontol.

[14] Chen Y, Wong RW, McGrath C, Hagg U, Seneviratne CJ (2014). Natural compounds containing mouthrinses in the management of dental plaque and gingivitis: a systematic review. Clin Oral Investig.

[15] Kaur H, Jain S, Kaur A (2014). Comparative evaluation of the antiplaque effectiveness of green tea catechin mouthwash with chlorhexidine gluconate. J Indian Soc Periodontol.

[16] Kumar SB (2017). Chlorhexidine mouthwash- a review. J pharmaceutical Sci Res.

[17] Emilson CG (1977). Susceptibility of various microorganisms to chlorhexidine. Scand J Dent Res.

[18] Greenstein G, Berman C, Jaffin R (1986). Chlorhexidine: an adjunct to periodontal therapy. J Periodontol.

[19] BD EDEN. Prevention strategies for periodontal diseases. In: Prevention in Clinical Oral Health Care: Elsevier; 2008. p. 213–29. https://www.sciencedirect.com/science/article/pii/B9780323036955500203.

[20] Hegazy SA, Awad SM. Antibacterial effect of plant extracts on plaque and saliva in a group of high caries risk children. Egypt Dent J. 2012;56(4) https://www.researchgate.net/publication/309705149.

[21] Jauhari D, Srivastava N, Rana V, Chandna P. Comparative evaluation of the effects of fluoride mouthrinse, herbal mouthrinse and oil pulling on the caries activity and Streptococcus mutans count using Oratest and Dentocult SM strip mutans kit. Int J Clin Pediatr Dent. 2015;8(2):114–8 Available from: https://www.ncbi.nlm.nih.gov/pmc/articles/PMC4562043/. 10.5005/jp-journals-10005-1295 PMC4562043 26379378

[22] Al-Dabbagh SA, Qasim HJ, Al-Derzi NA (2016). Efficacy of Miswak toothpaste and mouthwash on cariogenic bacteria. Saudi Med J.

[23] Narayan A, Mendon C. Comparing the effect of different mouthrinses on de novo plaque formation. J Contemp Dent Pract. 2012;13(4):460–3 PubMed PMID: 23151693. https://www.ncbi.nlm.nih.gov/pubmed/23151693 10.5005/jp-journals-10024-1169 23151693

[24] Bassiouny G, Al-Barrak H (2014). The anti-plaque effect of Miswak and myrrh mouthwashes versus chlorhexidine in the treatment of chronic gingivitis; a comparative clinical trial. Med Sci.

[25] Deshmukh MA, Dodamani AS, Karibasappa G, Khairnar MR, Naik RG, Jadhav HC (2017). Comparative evaluation of the efficacy of probiotic, herbal and chlorhexidine mouthwash on gingival health: a randomized clinical trial. J Clin Diagn Res.

[26] Rahmani ME, Radvar M (2005). The antiplaque effects of Salvadora persica and Padina essential oil solution in comparison to chlorhexidine in human gingival disease; a randomized placebo-controlled clinical trial. Int J Pharmacol.

[27] Vandana S, Supreet K, Tej SS, Neelam S, Simrat K, Reefat M (2014). Evaluation of clinical efficacy and safety of commercially available herbal mouthwash (HiOra)^R^ in comparison with chlorhexidine mouthwash (Aster-X)^R^ in improving oral health in patients undergoing dental procedures: a double blind, randomized, active-controlled. Bfudj..

[28] Al-Bayaty FH, Ai-Koubaisi AH, Abdul N, Ali W, Abdulla MA (2010). Effect of mouth wash extracted from Salvadora persica (Miswak) on dental plaque formation: a clinical trial. J Med Plants Res.

[29] Gupta R, Yadav OP, Khan M, Kaushik S, Ahmed N, Panwar M (2017). Comparative evaluation of efficacy of Hiora, Terminalia chebula and chlorhexidine as mouth wash on dental plaque. J Dent Health Oral Disord Ther.

[30] Prasad KA, John S, Deepika V, Dwijendra KS, Reddy BR, Chincholi S (2015). Anti-plaque efficacy of herbal and 0.2% chlorhexidine gluconate mouthwash: a comparative study. J Int Oral Health.

[31] Ghasemi M, Rahbar M, Valaei N (2014). Comparison of the Substantivity of several Mouthwashesand their effect on microbial plaque using epifluorescence microscope. J Islam Dent Assoc Iran.

[32] Bhat N, Mitra R, Oza S, Mantu VK, Bishnoi S, Gohil M (2014). The antiplaque effect of herbal mouthwash in comparison to chlorhexidine in human gingival disease: a randomized placebo controlled clinical trial. J Complement Integr Med.

[33] Bhate D, Jain S, Kale R, Muglikar S (2015). The comparative effects of 0.12% chlorhexidine and herbal oral rinse on dental plaque-induced gingivitis: a randomized clinical trial. J Indian Soc Periodontol.

[34] Singh A, Daing A, Dixit J (2013). The effect of herbal, essential oil and chlorhexidine mouthrinse on de novo plaque formation. Int J Dent Hyg.

[35] Abdulbaqi HR, Himratul-Aznita WH, Baharuddin NA (2016). Evaluation of Salvadora persica L. and green tea anti-plaque effect: a randomized controlled crossover clinical trial. BMC Complement Altern Med.

[36] Jaidka S, Somani R, Bajaj N, Jaidka R, Sharma S, Singh A (2015). Comparative evaluation of various mouthwashes for their effect on oral health : an in-vivo study. IJOCR..

[37] Schulz KF, Altman DG, Moher D, CONSORT Group (2010). CONSORT 2010 statement: updated guidelines for reporting parallel group randomised trials. BMJ.

[38] Higgins JP, Thompson SG, Deeks JJ, Altman DG (2003). Measuring inconsistency in meta-analyses. BMJ..

[39] Shekelle PG, Woolf SH, Eccles M, Grimshaw J (1999). Clinical guidelines: developing guidelines. BMJ..

[40] Liberati A, Altman DG, Tetzlaff J, Mulrow C, Gøtzsche PC, Ioannidis JP (2009). The PRISMA statement for reporting systematic reviews and meta-analyses of studies that evaluate health care interventions: explanation and elaboration. PLoS Med.

[41] Bagchi S, Saha S, Jagannath GV, Reddy VK, Sinha P (2015). Evaluation of efficacy of a commercially available herbal mouthwash on dental plaque and gingivitis: a double-blinded parallel randomized controlled trial. J Indian Assoc Public Health Dent.

[42] James P, Worthington HV, Parnell C, Harding M, Lamont T, Cheung A, Whelton H, Riley P (2017). Chlorhexidine mouthrinse as an adjunctive treatment for gingival health. Cochrane Database Syst Rev.

[43] Bonesvoll P, Lökken P, Rölla G (1974). Influence of concentration, time, temperature and pH on the retention chlorhexidine in the human oral cavity after mouth rinses. Arch Oral Biol.

